# The trans DNA cleavage activity of Cas12a provides no detectable immunity against plasmid or phage

**DOI:** 10.3389/fgeed.2022.929929

**Published:** 2022-07-26

**Authors:** Shunhang Liu, Xichen Rao, Ruiliang Zhao, Wenyuan Han

**Affiliations:** ^1^ State Key Laboratory of Agricultural Microbiology and College of Life Science and Technology, Hubei Hongshan Laboratory, Huazhong Agricultural University, Wuhan, China; ^2^ State Key Laboratory of Protein and Plant Gene Research, School of Life Sciences, Peking University, Beijing, China

**Keywords:** CRISPR-cas system, Cas12a (Cpf1), trans cleavage activity, plasmid targeting, anti-phage activity

## Abstract

Cas12a is a type V-A CRISPR-Cas RNA-guided endonuclease. It cleaves dsDNA at specific site, and then is activated for nonspecific ssDNA cleavage in trans *in vitro*. The immune function of the trans activity is still unknown. To address this question, we constructed a Cas12a targeting system in *Escherichia coli*, where Cas12a cleaved a high-copy target plasmid to unleash the trans ssDNA cleavage activity. Then, we analyzed the effect of the Cas12a targeting on a non-target plasmid and a ssDNA phage. The results show that Cas12a efficiently eliminates target plasmid but exerts no impact on the maintenance of the non-target plasmid or plague formation efficiency of the phage. In addition, a two-spacer CRISPR array, which facilitates target plasmid depletion, still has no detectable effect on the non-target plasmid or phage either. Together, the data suggest that the trans ssDNA cleavage of Cas12a does not contribute to immunity *in vivo*.

## Introduction

Cas12a, previously known as Cpf1, is the effector of type V-A CRISPR-Cas immune system (Zetsche et al., 2015; Paul and Montoya, 2020). It processes its CRISPR (cr) RNA and is directed by the crRNA to recognize and cleave target dsDNA in PAM-dependent manner (Zetsche et al., 2015; Fonfara et al., 2016), thus providing immunity against dsDNA phage and plasmid. This feature also endows Cas12a the ability for multiplex gene editing (Zetsche et al., 2017). The dsDNA cleavage activity is activated by the hybridization between crRNA and the target strand of dsDNA, which results in conformational changes and the exposure of the RuvC catalytic site (Swarts et al., 2017; Stella et al., 2018; Swarts and Jinek, 2019). Then, the RuvC successively cleaves the non-target strand and the target strand of dsDNA. After dsDNA cleavage, Cas12a still binds the complementary strand and preserves the active state for nonspecific ssDNA cleavage in trans (Li et al., 2018a; Chen et al., 2018). The activity has led to the development of Cas12a-based nucleic acid detection tools (Li et al., 2018b; Chen et al., 2018). However, the immune functions of the trans activity *in vivo* have not been investigated. Invading DNA, such as plasmids and phages, may provide ssDNA from their replication fork and transcription bubble. Some of plasmids and phages undergo ssDNA stage during their replication cycle. Thus, the trans activity may degrade such ssDNA and provide immunoprotection. On the other hand, the trans activity may also cleave ssDNA from genomic DNA and thus result in off-target effect when Cas12a is applied in genome editing. To address the concern, we programmed Cas12a to target a high-copy plasmid and evaluated the effects of the Cas12a on non-target plasmid and phage. We reveal that the Cas12a targeting does not provide immunity against non-target plasmid or phage.

## Materials and methods

### Bacteria strains and M13 phage

*Escherichia coli* DH5α was used to construct the plasmids used in the study. *E. coli* MG1655 and JM109 were used as the hosts to analyze plasmid maintenance and M13 infection respectively. The *E. coli* strains were grown in lysogeny broth (LB) medium at 37°C or 30°C as indicated. When required, antibiotics, including 100 μg/ml ampicillin (Amp) and/or 25 μg/ml chloramphenicol (Chl), were supplemented into the medium. The transformants containing pCas12a plasmids were grown in LB containing 0.2% glucose to suppress protein expression unless Cas12a was induced. When indicated, 5 mg/mL l-arabinose (L-ara), 80 ng/ml anhydrotetracycline (aTc) and 1 mM isopropylthio-β-galactoside (IPTG) were supplemented into the medium to induce protein and/or crRNA expression. M13 phage was a gift from Prof. Shi Chen.

### Construction of pCas12 and pCas9 plasmids and *E. coli* strains

The plasmids used to construct Cas12a targeting and Cas9 targeting includes p^TS^Cas12a-1S, p^TR^Cas12a-1S, p^TR^Cas12a-2S, p^TR^Cas12a-NT, p^TS^Cas9 and pTarget (pUC19) (Supplementary Table S1). The pCas12 plasmids were constructed using p46Cpf1 (Addgene: #98592) (Ao et al., 2018) and a synthesized CRISPR array. The CRISPR array containing two repeats interspaced by two SapI sites (Supplementary Table S3) was synthesized in a pCOLADuet vector backbone (Tingke, Beijing, China), i.e. pArray (Supplementary Table S1). We firstly generated the spacer sequence by annealing 1 S-F and 1S-R, and inserted the spacer sequence into the CRISPR array between the two SapI sites to obtain a CRISPR array targeting pTarget. Then, the CRISPR array and the p46Cpf1 backbone were amplified using Array-F/R and p46Cpf1-F/R primer sets, digested with XmaI and XhoI, and ligated to obtain p^TS^Cas12a-1S.

To generate the p^TR^Cas12 plasmids, the wild type pSC101 replicon was amplified from the pSB4k5 plasmid using TR-F/R primers, while the pCas12a fragments depletion of the origin and rep101 sequence were amplified from p^TS^Cas12a-1S. Then, the fragments were assembled using ClonExpress II One Step Cloning Kit (Vazyme, Nanjing, China) to obtain the temperature-resistant plasmids.

p^TR^Cas12a-2S and p^TR^Cas12a-NT were constructed in a similar way. The original CRISPR array with two SapI sites was used as the non-targeting (NT) array. We confirmed that the spacer does not match any sequence of *E. coli* genome or pTarget using blast analysis. The two-spacer array was generated by the golden gate assembly strategy (Li et al., 2020) using the four oligos listed in Supplementary Table S3. Then, the arrays were amplified and ligated with p46Cpf1 backbone as described above.

p^TS^Cas9 was constructed from the pCas plasmid (Addgene: #62225) (Jiang et al., 2015), which expresses a sgRNA targeting the pMB1 origin of pTarget. Specifically, two genetic modules were amplified from pCas using corresponding primers, including module1: replicon-lacI-P_trc_-sgRNA, and module2: tracrRNA-cas9. In addition, the Chl-resistant gene was amplified from p46Cpf1. The three fragments were assembled using ClonExpress II One Step Cloning Kit (Vazyme, Nanjing, China) to obtain p^TS^Cas9.

Each pCas12 or pCas9 plasmid was transformed in *E. coli* together with pTarget to generate the strains for further analysis. The strains include MG1655:p^TS^Cas12a-1S + pTarget, MG1655:p^TS^Cas9+pTarget, MG1655:p^TR^Cas12a-1S + pTarget, MG1655:p^TR^Cas12a-2S + pTarget, MG1655:p^TR^Cas12a-NT + pTarget, JM109:p^TR^Cas12a-1S + pTarget, JM109:p^TR^Cas12a-2S + pTarget, JM109:p^TR^Cas12a-NT + pTarget (Supplementary Table S2).

All the primers used to construct the plasmids are listed in Supplementary Table S3.

### Plasmid maintenance assay

The strains were inoculated from glycerol stock to 5 ml LB medium containing corresponding antibiotics. The cultures were grown for overnight at 37° C (temperature-resistant plasmids) or 30°C (temperature-sensitive plasmids), 10 μL of which was transferred into 10 ml fresh antibiotics-free medium with indicated inducers. Specifically, Cas12a was induced by 5 mg/mL L-ara, crRNA was induced by 80 ng/ml aTc, while the transcription of sgRNA from p^TS^Cas9 was initiated by 1 mM IPTG. At indicated time post induction, the cells were serially diluted and dropped onto plates containing 100 μg/ml Amp or 25 μg/ml Chl or both, as well as antibiotics-free plates. The plates were incubated at 37°C or 30°C for overnight, and colonies were counted. Relative colony formation units (CFU) were calculated using CFU of antibiotics-free plates as the reference.

### qPCR

The cells containing p^TS^Cas12a-1S and pTarget were grown in the medium with either L-ara and aTc (Cas12a targeting) or only L-ara (control) in the absence of any antibiotics at 30°C. At 6 h post induction, the cultures were diluted by 1000 times with water and heated at 95°C for 10 min. The heated cell suspension was directly used as template for qPCR assay using Hieff UNICON Universal Blue qPCR SYBR Green Master Mix (Yeasen Biotechnology, Shanghai, China) following the protocol provided by the manufacture. The cycle threshold (Ct) values were used to define the abundance of each plasmid with the *E. coli* 16s rDNA as the reference gene. The relative plasmid abundance was calculated by the abundances of p^TS^Cas12a-1S and pTarget in the Cas12a targeting culture (L-ara and aTc) normalized to those in the control culture (only L-ara). The primers for qPCR are listed in Supplementary Table S3.

### Phage plaque formation assay

*E. coli* JM109 strains were inoculated from a single colony to 5 ml LB medium containing corresponding antibiotics. The cultures were grown for overnight at 37°C, 50 μL of which was transferred into 5 ml antibiotics-free medium and further grown at 37°C for 4 h. Then, 200 μL of the culture was mixed with 4 ml of 0.7% top layer agar and spread on LB-agar plates. Both top layer agar and LB-agar plate contain 5 mg/mL L-ara and 80 ng/ml aTc to initiate Cas12a targeting. After the plates were solidified, serially diluted M13 phage solutions were dropped on to the plates. The plates were incubated at 37°C for 36 h, and then pictured.

### Phage replication assay

500 μL of *E. coli* JM109 cultures post overnight growth were transferred into 50 ml fresh medium without antibiotics and further grown for 1 h. Then, the cultures were diluted to ∼1×10^8^ cell/mL and cooled on ice. M13 phage was supplemented into the cultures at an multiplicity of infection (MOI) of about 0.1. The resulting mixtures were incubated at 4°C for 5 min, and then the cells were collected by centrifugation at 3000 *g* for 10 min and the supernatant was removed. The cells were resuspended by 50 ml pre-warmed fresh medium containing 5 mg/mL L-ara and 80 ng/ml aTc and grown at 37°C. At 30, 60, 90, 120 min post induction, aliquots of the cultures were sampled, cells were removed by centrifugation, and the viral titers in the supernatant were calculated using plaque formation assay as described above.

## Results

**FIGURE 1:**
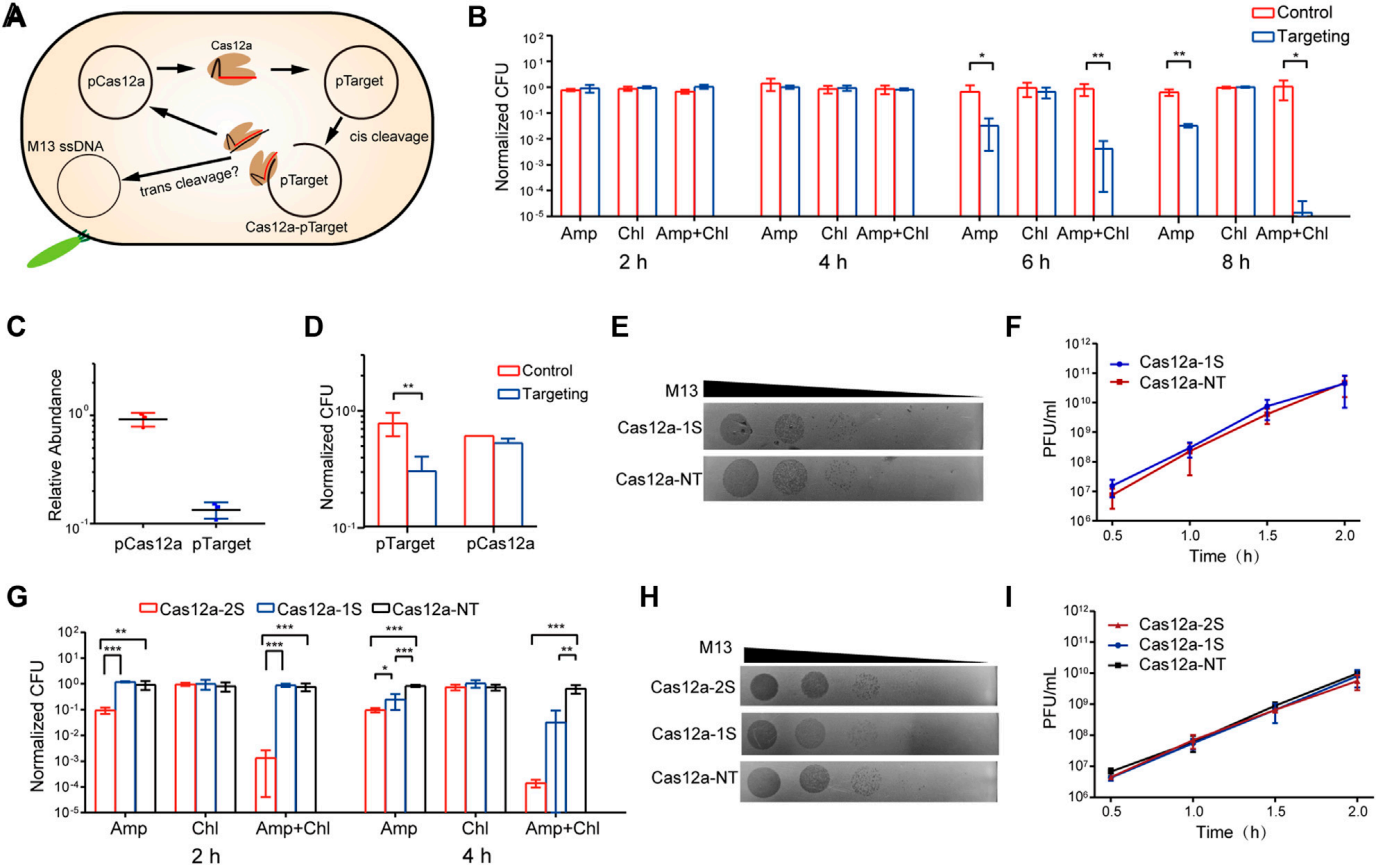
Cas12a trans activity does not provide immunity against plasmid or phage **(A)** An illustration of the design of the study. The pCas12a plasmid expresses Cas12a complex, which is directed by crRNA to cleave pTarget. The cleavage generates Cas12a-crRNA-target DNA complex, the effects of which on pCas12a or M13 phage are analyzed **(B)** Plasmid maintenance of pTarget (Amp-resistant) and p^TS^Cas12a-1S (Chl-resistant) after initiating Cas12a targeting. Normalized CFU was calculated using the CFU on the antibiotics-free plates as reference **(C)** qPCR analysis of the relative abundance of pTarget and p^TS^Cas12a-1S after initiating Cas12a targeting **(D)** Plasmid maintenance of pTarget and p^TS^Cas12a-1S after initiating Cas12a targeting at 37°C **(E)** Plague formation assay of M13. Ten-fold serial dilutions of M13 were dropped onto bacterial lawns containing p^TR^Cas12a-1S or p^TR^Cas12a-NT **(F)** M13 titers after infecting cells containing p^TR^Cas12a-1S or p^TR^Cas12a-NT **(G)** Plasmid maintenance of pTarget (Amp-resistant) and pCas12a plasmids (Chl-resistant) in cells containing p^TR^Cas12a-2S, p^TR^Cas12a-1S and p^TR^Cas12a-NT respectively **(H)** Plague formation assay of M13. Ten-fold serial dilutions of M13 were dropped onto bacterial lawns containing p^TR^Cas12a-2S, p^TR^Cas12a-1S and p^TR^Cas12a-NT respectively **(I)** M13 titers after infecting cells containing p^TR^Cas12a-1S, p^TR^Cas12a-1S or p^TR^Cas12a-NT. All the quantification data are shown as means of three independent replicates. Error bars indicate the standard deviations. The *p*-values were calculated by single tail Student’s *t* Test. *: *p* < 0.05, **: *p* < 0.01, ***: *p* < 0.001.

We chose *Francisella novicida* Cas12a to analyze the immune function of Cas12a trans activity, which has been shown to cleave ssDNA in trans *in vitro* (Li et al., 2018a) and widely applied in genome editing in both bacteria and eukarya (Endo et al., 2016; Jiang et al., 2017; Tu et al., 2017). For this purpose, we constructed a Cas12a targeting system in *Escherichia coli* MG1655 (Figure 1A and Supplementary Figure S1). The system contains two plasmids. The p^TS^Cas12a-1S plasmid expresses Cas12a protein using an ara promoter (P_ara_), and a crRNA under a tet promoter (P_tet_). The crRNA targets the *amp* gene of pUC19, which serves the target plasmid (pTarget). Upon addition of the inducers (L-ara and aTc), Cas12a and crRNA are expressed and form a ribonucleoprotein complex to cleave pTarget, generating Cas12a-crRNA-target DNA complex, which is active for trans ssDNA cleavage. The active complex might degrade p^TS^Cas12a-1S, the non-target plasmid; therefore, we can evaluate the trans effect of Cas12a targeting by measuring the stability of p^TS^Cas12a-1S. Notably, the copy numbers of p^TS^Cas12a-1S and pTarget are ∼5 and 500-700, respectively. The abundant pTarget plasmid should provide enough substrate to produce high level of Cas12a-crRNA-target DNA complex *in vivo*.

We initiated Cas12a targeting in antibiotics-free medium by supplementing L-ara and aTc, while the culture only supplemented with L-ara to avoid crRNA expression was set as control. Then, the plasmid maintenance was analyzed by measuring the colony formation units (CFU) on the plates containing corresponding antibiotics. The relative CFU were normalized to those on the antibiotics-free plates. The results show that Cas12a targeting reduced Amp-resistant CFU by ∼10-fold from 4 to 6 h post induction, indicative of pTarget depletion, while the maintenance of p^TS^Cas12a-1S (Chl-resistant) was not affected during the time (Figure 1B and Supplementary Figure S2). In addition, the CFU resistant to both Amp and Chl was dramatically reduced by 10^3^ and 10^5^ folds at 6 and 8 h post induction respectively. To investigate whether this phenomenon is related to the trans activity of Cas12a, we constructed a Cas9 targeting system and analyzed its effects on plasmid maintenance. Cas9 mediates dsDNA targeting, but does not have trans ssDNA cleavage (Jinek et al., 2012). The results show that Cas9 targeting also resulted in a dramatic reduction of the CFU resistant to both antibiotics, as well as a moderate loss of pTarget (Amp-resistant) (Supplementary Figure S3). The similar performance of Cas9 system and Cas12a system suggests that the Amp-resistant colonies could be a consequence of spontaneous loss of p^TS^Cas12a-1S or p^TS^Cas9, instead of the trans cleavage of the plasmids, as evidenced by the equal stability of p^TS^Cas12a-1S and p^TS^Cas9 plasmids in both targeting systems.

To investigate whether the trans activity of Cas12a would reduce the copy number of the non-target plasmid, we analyzed the relative abundance of p^TS^Cas12a-1S and pTarget using qPCR after initiating Cas12a targeting. We set the abundance of pTarget and p^TS^Cas12a-1S in the control culture as 1. The results show that Cas12a targeting reduced the pTarget abundance by about 7-fold, but exerted no effect on the p^TS^Cas12a-1S abundance (Figure 1C).

To exclude that the replication of the plasmid might have compromised the effects of Cas12a trans activity, we initiated Cas12a targeting at 37°C, at which temperature the temperature-sensitive replicon cannot replicate the plasmid. Growing at 37°C resulted in p^TS^Cas12a-1S depletion in ∼40% cells in the control culture (Figure 1D), in agreement with the replication inhibition. However, Cas12a targeting did not lead to further p^TS^Cas12a-1S depletion. In contrast, Cas12a targeting reduced pTarget-containing cells by ∼2.6 fold (Figure 1D). The lower pTarget depletion efficiency could be due to faster spontaneous loss of p^TS^Cas12a-1S at 37°C.

Next, we wanted to analyze whether the trans activity of Cas12a would confer immunity against the ssDNA phage M13. For this purpose, we constructed two temperature-resistant plasmids: p^TR^Cas12a-1S and p^TR^Cas12a-NT, the latter of which expresses a crRNA that does not match any sequence of pTarget or *E. coli* genome and was set as control. The plasmids were transformed into *E. coli* JM109 together with pTarget, respectively. Comparison of plasmid maintenance in the resulting strains indicates that Cas12a targeting led to depletion of pTarget not p^TR^Cas12a-1S (Supplementary Figure S4A), in line with the analysis in MG1655. Then, we analyzed the susceptibility of the strains to M13 using plague formation units (PFU) assay. The assay did not reveal any difference between the two strains. To further analyze the effect of Cas12a targeting on M13 propagation, we infected the strains in liquid culture and initiated Cas12a targeting by supplementing the inducers at the same time. Since Cas12a targeting mainly eliminated pTarget in the 2 h post induction (Supplementary Figure S4B), we measured the phage titers during this time. The data reveal the same M13 titers from the two strains, indicating that Cas12a targeting exerted no effect on M13 propagation (Figure 1F).

After cleavage of the target dsDNA, Cas12a complex is still attached to the target strand. We assumed that the attachment might limit the mobility of Cas12a complex and thus restrict the trans cleavage activity. To release the Cas12a-crRNA-target DNA complex, we designed a two-spacer CRISPR array (Supplementary Figure S1B). The two spacers match two adjacent protospacers of pTarget, and thus cleavage of one copy of pTarget will generate two active Cas12a complexes, one of which only binds a short fragment of target DNA and is supposedly more mobile. We compared the maintenance of pTarget and non-target plasmids (pCas12 plasmids) in the cells containing one spacer (1 S), two spacers (2 S) and a non-target spacer, respectively. The results show that Cas12a induced pTarget depletion in the presence of both one and two spacers, and that the 2 S system more efficiently eliminated pTarget than the 1 S system. However, neither the 2 S nor the 1 S system affected the maintenance of pCas12a (Figure 1G). Further, we analyzed the M13 susceptibility of the *E. coli* JM109 cells containing the 2 S system and M13 propagation in the cells containing the 2 S system. Again, the results show that the 2 S system did not induce any reduction in M13 PFU or M13 titers compared to the control culture, either (Figures 1H,I). Collectively, the data indicate that Cas12a, when activated by target dsDNA cleavage, cannot provide immunity against non-target plasmid or phage.

## Discussion

In this study, we constructed a Cas12a targeting system by programming it to target a high-copy plasmid in *E. coli*. As indicated by the *in vitro* studies (Li et al., 2018a; Chen et al., 2018), cleavage of the target plasmid triggers the trans nonspecific ssDNA cleavage activity of Cas12a. However, our data suggest that the trans activity dose not play a detectable immune function against plasmid or phage. The phenomena may be caused by several reasons. First, the abundant ssDNA binding proteins *in vivo* can protect ssDNA from active Cas12a. Second, active Cas12a could be rapidly reset by replacement of crRNA-target DNA hybrid with new crRNAs (Stella et al., 2018). In addition, the host used in this study, *E. coli*, may lack essential factors that are required for Cas12a to achieve full functions. Therefore, we cannot deny that the trans activity of Cas12a may play immune and/or other functions in its native host.

Cas12a has been widely used as genome editing tools. A major concern of its application is the potential off-targeting. Previous reports have indicate the high specificity of Cas12a in mammals (Kim et al., 2016; Kleinstiver et al., 2016; Wei et al., 2021). In *E. coli*, we demonstrate that even in the presence of 500-700 copies of target and two spacers, Cas12a did not cause apparent trans cleavage. Most recently, an independent study on Lachnospiraceae *bacterium* (Lb) Cas12a also reveals that the trans cleavage does not play a detectable role in immunity (Marino et al., 2022). Together, the data indicate that Cas12a is an accurate nuclease when used for gene editing, plasmid elimination and anti-phage in bacteria.

## Data Availability

The original contributions presented in the study are included in the article/Supplementary Material, further inquiries can be directed to the corresponding author.
