# Supplementary material for: The trans DNA cleavage activity of Cas12a provides no detectable immunity against plasmid or phage
Source: Front Genome Ed. 2022 Jul 26;4:929929. doi: 10.3389/fgeed.2022.929929 (PMC9360544; doi:10.3389/fgeed.2022.929929)
Supplement: Supplementary file 1 [file DataSheet1.PDF]

Supplementary figures

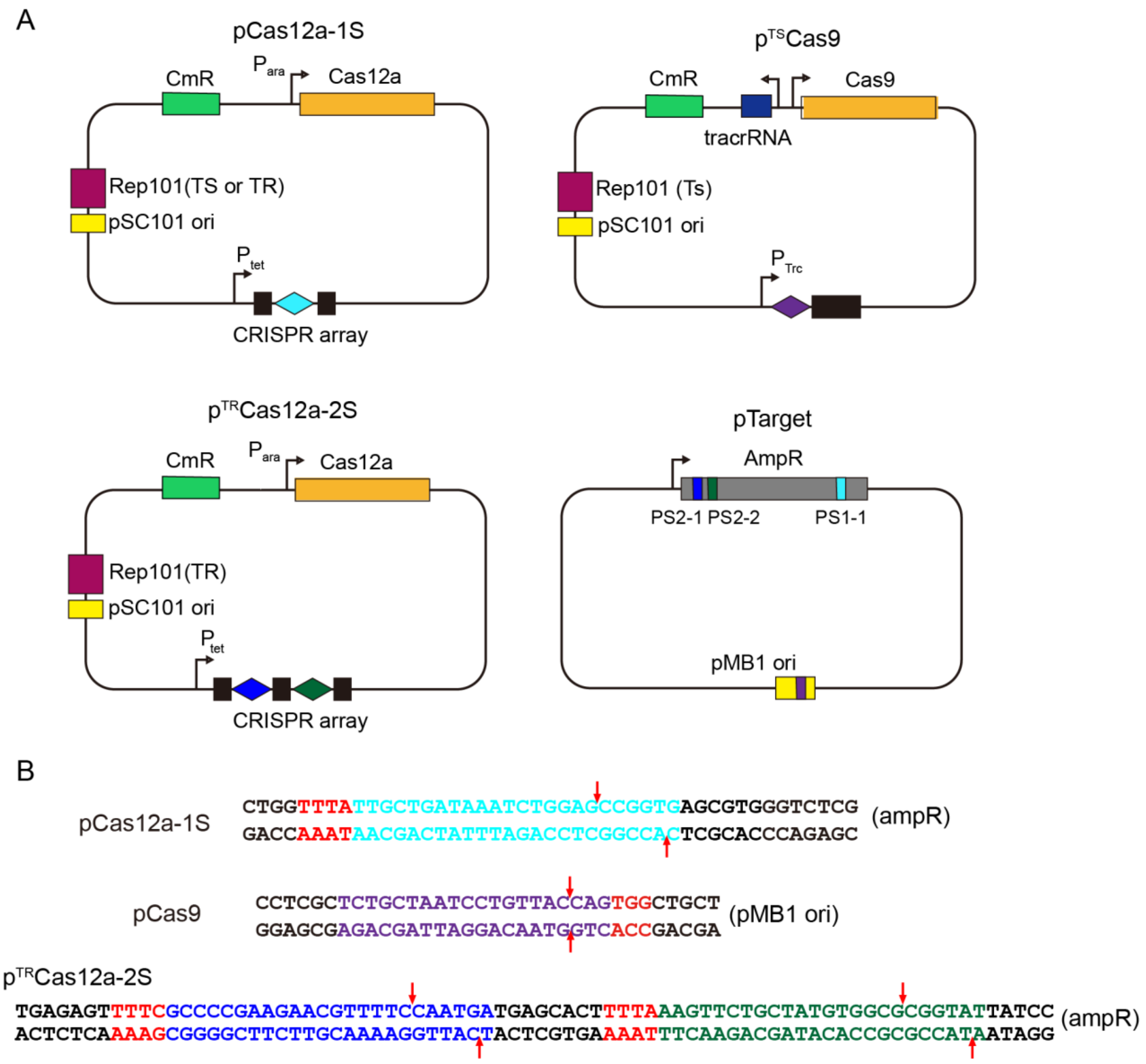

Figure S1 (A) Schematic of the plasmids to generate Cas12a or Cas9 targeting. The plasmids include p^{TS}Cas12a-1S, p^{TR}Cas12a-1S, p^{TS}Cas9, p^{TR}Cas12a-2S and pTarget. The protopsacers (PS) in pTarget are indicated with the same color as the spacers. (B) An illustration of the cleavage of pTarget by each pCas plasmid. The protopsacers are indicated with the same color as in (A), while PAM is shown in red. The cleavage sites are marked with red arrows. The genetic elements containing the protospacers are also indicated.

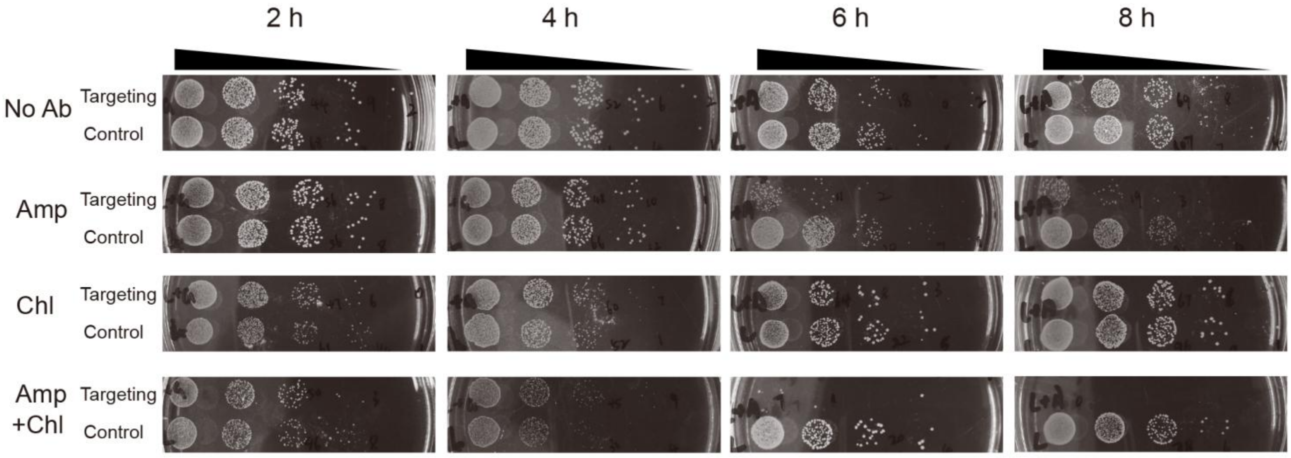

Figure S2 Cas12a targeting resulted in depletion of pTarget (Amp-resistant) not p^{TS}Cas12a-1S (Chl-resistant). In the MG1655::p^{TS}Cas12a-1S+pTarget strain, Cas12a targeting was initiated by supplementing L-ara and aTc. At 2, 4, 6 and 8 h post induction, the cells were serially diluted and dropped onto plates containing indicated antibiotics. Control: Cas12a targeting was suppressed by only supplementing L-ara. No Ab: antibiotics-free plates. Images show results of three independent replicates.

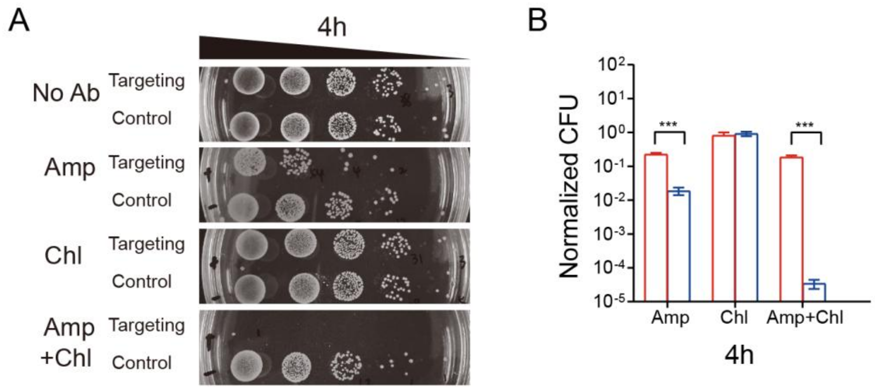

Figure S3 Effects of Cas9 targeting on plasmid maintenance. Cas9 targeting was induced by supplementing IPTG, and the culture in the absence of IPTG was set as control. At 4 h post induction, the cells were serially diluted and dropped onto plates containing indicated antibiotics. (A) A representative image of three independent replicates. (B) Quantification of the results from (A). The data show means of three independent replicates. Error bars indicate the standard deviations. The p-values were calculated by single tail Student's T Test. ***: $p<0.001$.

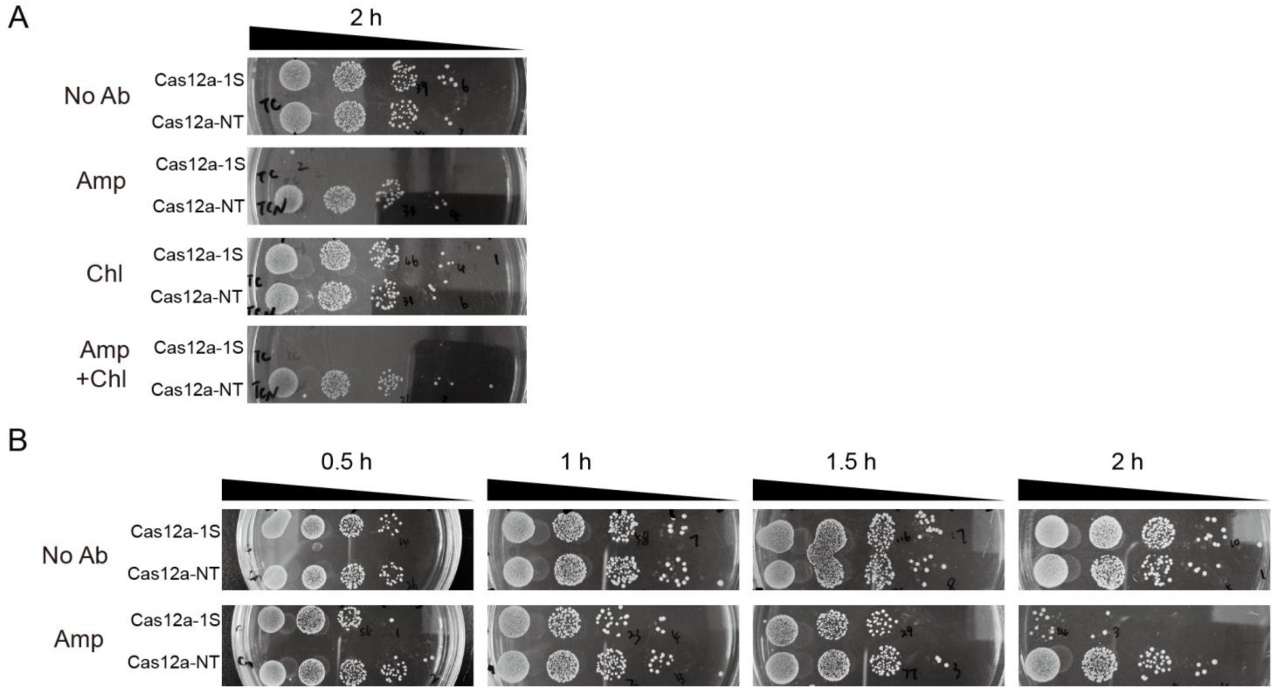

Figure S4 Cas12a targeting resulted in pTarget depletion in JM109 at 37 ^{o}C. (A) The JM109 cultures containing p^{TR}Cas12a-1S or p^{TR}Cas12a-NT were supplemented with L-ara and aTc. At 2 h post induction, the cells were serially diluted and dropped onto the plates containing indicated antibiotics. No Ab: antibiotics-free plates. (B) The cells were dropped onto antibiotics-free and amp plates at different times post induction.

Supplementary tables

Table S1. Plasmids used in the study.

Plasmids	Description
**Plasmids used for protein expression in *E.coli***	
p46Cpf1	Purchased from Addgene (#98592) (1)
pArray	pCOLADuet containing two repeats separated by two SapI sites
p^{TS}Cas12a-1S	Derived from p46Cpf1, containing the *cas12a* gene and a spacer targeting the *amp* gene. Temperature-sensitive origin
pTarget	pUC19
pCas	A gift from Shan Li lab, Addgene: #62225 (2)
p^{TS}Cas9	Derived from p^{TS}Cas9, expressing Cas9 and sgRNA targeting pMB1 origin of pTarget. Temperature-sensitive origin
pSB4k5	Containing wild type pSC101 orign and *rep101* gene, iGEM Foundation
p^{TR}Cas12a-1S	Derived from p46Cpf1, containing the *cas12a* gene and a spacer targeting the *amp* gene. Temperature-resistant origin
p^{TR}Cas12a-2S	Derived from p46Cpf1, containing the *cas12a* gene and two spacers targeting the *amp* gene. Temperature-resistant origin
p^{TR}Cas12a-NT	Derived from p46Cpf1, containing the *cas12a* gene and a spacer without any target sequence in *E. coli* genome or pUC19. Temperature-resistant origin

Table S2. Strains constructed in the study.

**Strains**	**Description**
MG1655::p^{TS}Cas12a-1S+pTarget	Used for plasmid maintenance assay (Figure1B, C and D, Figure S2)
MG1655::p^{TS}Cas9+pTarget	Used for plasmid maintenance assay (Figure S3)
MG1655::p^{TR}Cas12a-2S+pTarget	Used for plasmid maintenance assay (Figure 1G)
MG1655::p^{TR}Cas12a-1S+pTarget	Used for plasmid maintenance assay (Figure 1G)
MG1655::p^{TR}Cas12a-NT+pTarget	Used for plasmid maintenance assay (Figure 1G)
JM109::p^{TR}Cas12a-1S+pTarget	Used for plasmid maintenance assay and M13 infection assay (Figure 1E, F and H, Figure S4)
JM109::p^{TR}Cas12a-2S+pTarget	Used for plasmid maintenance assay and M13 infection assay (Figure 1H)
JM109::p^{TR}Cas12a-NT+pTarget	Used as control for plasmid maintenance assay and M13 infection assay (Figure 1E, F and H, Figure S4)

Table S3. Synthesized DNA in the study.

**Name**	**Sequence**	**Description**
p46Cpf1-F	TCCCCCCGGGATTTCATGAAGTTCC	Amplification of Cas12 expression cassette, origin and *cam* from p46Cpf1 plasmid
p46Cpf1-R	CCGCTCGAGCCTTTCTCCTCTTTAG	
Array-F	CCGCTCGAGCAGGAAACAGCTGTCTAAGAAC	Amplification of CRISPR array from pArray
Array-R	TCCCCCCGGGATTATGCGGCCGCTC	
1S-F	GATTTGCTGATAAATCTGGAGCCGGTGAGCGTG	To generate a spacer by annealing
1S-R	GACCACGCTCACCGGCTCCAGATTTATCAGCAA	
2S-1F	GATGCCCCGAAGAACGTTTTCCAATGAGTCTAAGAACTTTAAATAATTTCTAC	To generate two spacers by annealing
2S-1R	AAATTATTTAAAGTTCTTAGACTCATTGGAAAACGTTCTTCGGGGC	
2S-2F	TGTTGTAGATAAGTTCTGCTATGTGGCGCGGTAT	
2S-2R	GACATACCGCGCCACATAGCAGAACTTATCTACAACAGTAG	
TR-F	TCAGATCCTTCCGTATTTAGCC	Amplification of wild type pSC101 origin and *rep101* from pSB4k5
TR-R	ATGTCTGAATTAGTTGTTTTCAAAGC	
p^{TS}Cas12a-1S-F	AAAACAACTAATTCAGACATACATCTCA ATTGGTCTAGGTG	Amplification of the fragment depletion of temperature-sensitive pSC101 origin and *rep101* from p^{TS}Cas12a
p^{TS}Cas12a-1S-R	CTAAATACGGAAGGATCTGAGGTTCTTAT GGCTCTTGTATCTATC	
CmRF	TTACGCCCCGCCCTGCC	Amplification of Chl-resistant gene from p46Cpf1
CmRR	ATGGAGAAAAAAATCACTGGATATACCA CC	
pCas-Module1-F	TATTATGACAACTTGACGGCCAATGGCGA TGACGCATC	Amplification of module1 from pCas
pCas-Module1-R	AGTGGCAGGGCGGGGCGTAATCAGAATT GGTTAATTGGTTGGAAAC	
pCas-Module2-F	CCAGTGATTTTTTTCTCCATAACACCCCT TGTATTACTGTTTATG	Amplification of module2 from pCas
pCas-Module2-R	GCCGTCAAGTTGTCATAATAAATCG	
q-16S-F	ATGTGGTTTAATTCGATGCAACGCG	qPCR analysis of 16S rDNA
q-16S-R	TGCGGGACTTAACCCAACATTTCAC	
q-pCas12a-F	ACTATCCCATATCACCAGCTCACCG	qPCR analysis of pCas12a
q-pCas12a-R	GACCGTTCAGCTGGATATTACGGCC	
q-pTarget-F	GGGATCATGTAACTCGCCTTGATCG	qPCR analysis of pTarget
q-pTarget-R	ATTGTTGCCGGGAAGCTAGAGTAA G	
Synthesized artificial CRISPR array	GTCTAAGAACTTTAAATAATTTCTACTGTTGTAGATGGAAGAGCCCGAGCTCTTCGGTCTAAGAACTTTAAATAATTTCTACTGTTGTAGAT	

1. Ao, X., Yao, Y., Li, T., Yang, T.T., Dong, X., Zheng, Z.T., Chen, G.Q., Wu, Q. and Guo, Y. (2018) A Multiplex Genome Editing Method for Escherichia coli Based on CRISPR-Cas12a. *Frontiers in microbiology*, **9**, 2307.
2. Jiang, Y., Chen, B., Duan, C., Sun, B., Yang, J. and Yang, S. (2015) Multigene editing in the Escherichia coli genome via the CRISPR-Cas9 system. *Applied and environmental microbiology*, **81**, 2506-2514.
